# A novel system for intensive *Diadema antillarum* propagation as a step towards population enhancement

**DOI:** 10.1038/s41598-021-90564-1

**Published:** 2021-05-27

**Authors:** Aaron R. Pilnick, Keri L. O’Neil, Martin Moe, Joshua T. Patterson

**Affiliations:** 1 School of Natural Resources and Environment, University of Florida/IFAS, 103 Black Hall, Gainesville, FL 32611 USA; 2 Center for Conservation, The Florida Aquarium, 529 Estuary Shore Lane, Apollo Beach, FL 33572 USA; 3 School of Forest, Fisheries, and Geomatics Sciences, University of Florida/IFAS, 7922 NW 71st Street, Gainesville, FL 32603 USA

**Keywords:** Ecosystem ecology, Restoration ecology

## Abstract

The long-spined sea urchin *Diadema antillarum* was once an abundant reef grazing herbivore throughout the Caribbean. During the early 1980s, *D. antillarum* populations were reduced by > 93% due to an undescribed disease. This event resulted in a lack of functional reef herbivory and contributed to ongoing ecological shifts from hard coral towards macroalgae dominated reefs. Limited natural recovery has increased interest in a range of strategies for augmenting herbivory. An area of focus has been developing scalable ex situ methods for rearing *D. antillarum* from gametes. The ultimate use of such a tool would be exploring hatchery origin restocking strategies. Intensive ex situ aquaculture is a potentially viable, yet difficult, method for producing *D. antillarum* at scales necessary to facilitate restocking. Here we describe a purpose-built, novel recirculating aquaculture system and the broodstock management and larval culture process that has produced multiple *D. antillarum* cohorts, and which has the potential for practical application in a dedicated hatchery setting. Adult animals held in captivity can be induced to spawn year-round, with some evidence for annual and lunar periodicity. Fecundity and fertilization rates are both consistently very high, yet challenges persist in both late stage larval development and early post-settlement survival. Initial success was realized with production of 100 juvenile *D. antillarum* from ~ 1200 competent larvae. While the system we describe requires a significant level of investment and technical expertise, this work advances *D. antillarum* culture efforts in potential future hatchery settings and improves the viability of scalable ex situ production for population enhancement.

## Introduction

Coral reef ecosystems are declining worldwide at alarming rates due to a variety of additive local and global environmental threats including coastal pollution, disease, climate change, and the loss of herbivores^1–3^. This decline challenges existing socio-ecological frameworks where human well-being relies on healthy and functional coral reef ecosystems^4^. Coral reefs provide an estimated $29.8 billion in global annual net benefits from economic activities and resources including fisheries, coastal shoreline protection, tourism, biodiversity, and biomedical applications^5–7^. Addressing coral reef decline fundamentally requires climate change and coastal pollution mitigation on a global scale. Nonetheless, resource managers are actively seeking novel strategies at local scales to address these rapid declines and to augment conservation efforts in hopes of preserving valuable biodiversity and ecosystem services^8^.

Marine conservation paradigms, which have traditionally focused on regulating human behavior to protect ecosystems and promote natural recovery (e.g. marine protected areas, catch limits and moratoriums, direct and non-point source pollution restrictions), are rapidly expanding to include restoration strategies involving direct intervention^4,9,10^. This expansion is evident in the Caribbean and Western-Atlantic, where unprecedented declines and lack of natural recovery of key reef-building corals^2,11,12^ have driven the rapid growth of propagation and restoration programs^13–17^. These restoration programs employ “coral gardening”^18^, to produce biomass from wild-collected fragments within in situ nurseries and outplant nursery-reared corals to degraded reefs^15,19–21^. In situ nurseries have functioned as genetic repositories^22^ and multi-year outplanting programs have been documented to increase coral abundance relative to un-restored reefs^23^. Simply outplanting propagated corals, however, does not address any of the stressors that led to reef decline and long term outplant survival can be low^24^. Practically, coral gardening should exist within a larger restoration framework that aims to reestablish functional natural reef structure and biodiversity via a multi-niche ecological approach.

Key to this ecological approach in the Caribbean and Western-Atlantic is the re-establishment of functional reef herbivory via recovery of long-spined sea urchin, *Diadema antillarum,* populations. As the primary generalist herbivore and bioeroder native to Caribbean reef ecosystems^25–27^, these large-bodied urchins historically maintained hardbottom in a state that favored stony coral recruitment and growth^28–30^. Historically, high population densities averaging 5–10 individuals m^−2^ were associated with low macroalgae cover, high hard coral cover, and high levels of habitat complexity^31,32^. In 1983–1984, *D. antillarum* populations were reduced by 93–100% throughout their native range following the spread of an undescribed disease^33–39^. This mass mortality event pervasively altered reef ecosystem dynamics via reduced herbivory and subsequent loss of hard coral cover, habitat complexity, and biodiversity^1,40–43^. To date, natural recovery has been extremely limited throughout most historical geographic ranges, with reported population densities averaging fewer than 0.3 individuals m^−2^ ^31^. It is widely acknowledged that the loss of functional herbivory from *D. antillarum* has strongly contributed to ongoing coral reef decline^44,45^.

**Figure 1:**
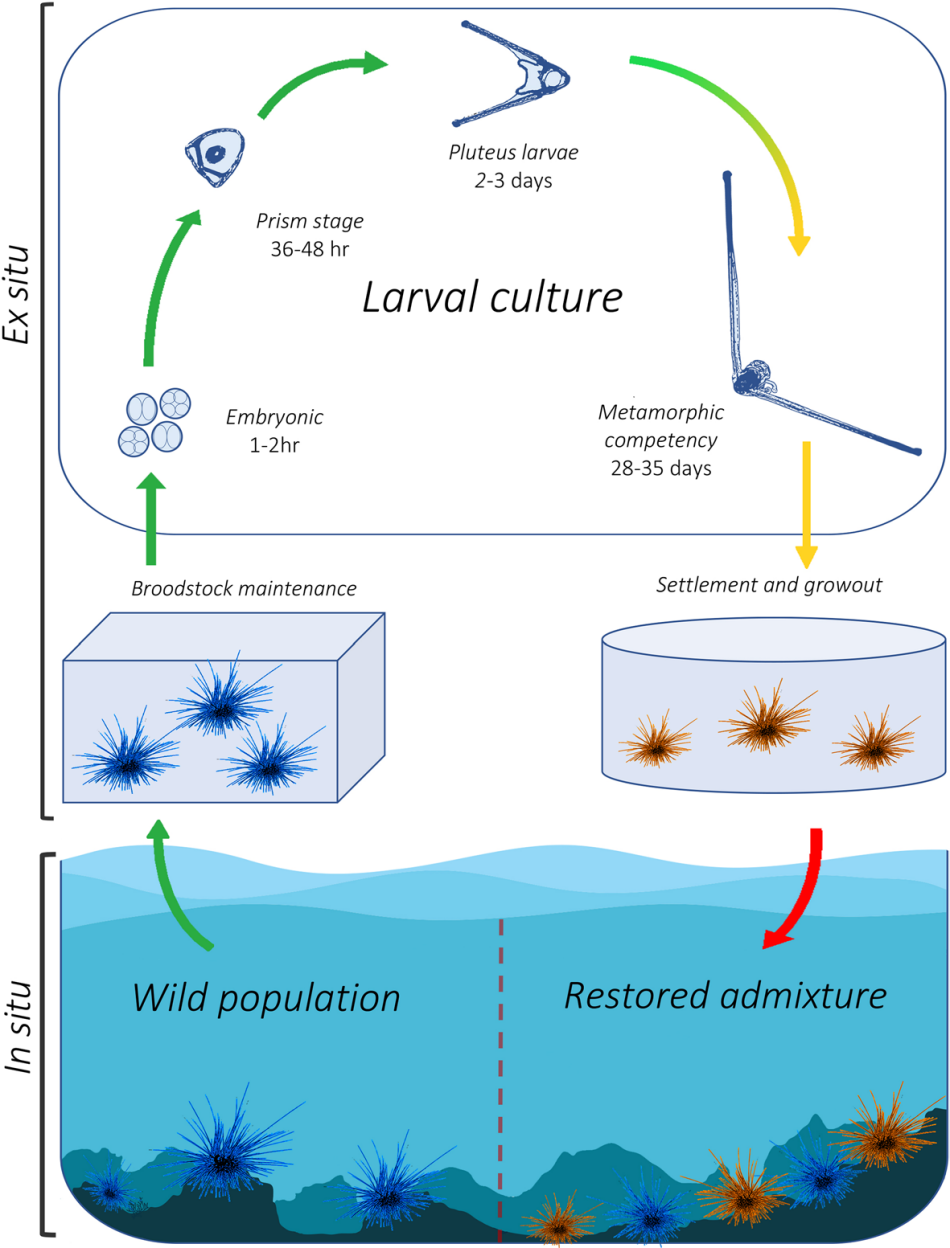
Conceptual model illustrating *D. antillarum* restocking via hatchery produced animals. Stages include in situ broodstock collection from wild populations, ex situ broodstock maintenance, larval development within scalable hatchery production settings, settlement and juvenile growout, and in situ restocking on targeted coral reefs. Arrow colors depict current levels of success in achieving each stage; green = achieved reliably at large scale, yellow = achieved somewhat reliably at reduced scale, red = not yet tested. The transition from green to yellow depicts a shift from reliable, successful production of hundreds of thousands of late-stage larvae to relatively fewer metamorphically competent larvae at 28–35 DPF (days post fertilization). Figure by Joseph A. Henry.

Coral reef managers are therefore highly interested in augmenting *D. antillarum* populations to restore functional herbivory and concurrently improve habitat for the recovery of key reef-building coral taxa^46^. These objectives would benefit from developing scalable ex situ methods for rearing this species from gametes^47^ and restocking hatchery-reared urchins to the wild (Fig. 1). While *D. antillarum* has been cultured successfully^48–50^, a lengthy and challenging larval development process, paucity of established culture methods, and lack of applicable commercial technologies has precluded the development of reliable production at restoration relevant scales. Here we describe a novel *D. antillarum* culture system designed to facilitate experimental research aimed at addressing fundamental knowledge gaps while developing practical and scalable production methods for restoration. A successful rearing protocol that has produced multiple hatchery cohorts is outlined. Additional information including broodstock management protocols, successful spawning records, and a timeline of development progression is also provided. Such information will be vital to advance captive propagation of this foundational species.

## Materials and methods

The objective of this study was to develop successful rearing protocols for *D. antillarum* from gametes through larval settlement in a novel culture system with the potential for scalable production. Hypothesis-driven experimentation examining larval survival and growth in response to microalgae diet combinations, diet concentrations, and initial larval stocking densities took place concurrent to this work. For the purposes of this report, only the best performing treatments from each of these parameters are included in the below methods.

### Broodstock maintenance

Adult *D. antillarum* broodstock were collected from patch reefs at ≤ 8 m depth off Marathon, Florida by the Florida Fish and Wildlife Research Institute under Florida Keys National Marine Sanctuary permit # FKNMS-2018-023 in March 2018. These animals were transported to shore in seawater-filled coolers and held temporarily in tanks with natural seawater. On 26 March 2018, 18 animals were transported in aerated, seawater-filled coolers to a land-based restoration aquaculture facility operated by The Florida Aquarium in Apollo Beach, FL. These urchins were quarantined in an enclosed greenhouse for a 45-day period, during which visual health assessments were performed prior to introduction to established holding systems. During quarantine, animals were fed daily and maintained within the temperature range described below for long-term holding. Some individuals arrived in Apollo Beach presenting tissue and spine loss and were treated under the direction of a veterinarian with oxytetracycline hydrochloride baths (15 mg L^−1^ every other day for 1–2 h, 3 doses) until their condition improved.

Following quarantine, 14 surviving broodstock were transferred to 450-L fiberglass tanks within a 2380-L recirculating aquaculture system (RAS) designed and concurrently used to house Caribbean corals. System life support included mechanical, biological, and chemical filtration through the use of a protein skimmer, live rock, 150-micron filter socks, and activated carbon. Water temperatures were maintained between 23.5 and 28.8 °C. Photoperiod varied naturally at the greenhouse latitude of N27° 46′ 43.81″. Nylon mesh was installed over portions of the tanks to provide shading (approximately 70% shading of incident light in greenhouse). Salinity was maintained at 34–37 g L^−1^ using artificial seawater (ASW) prepared from reverse osmosis deionized freshwater and a commercial salt mixture (Tropic Marin, Wartenburg, Germany). Broodstock were fed a commercially available herbivore diet ([34% crude protein, 8% crude fat, 8% crude fiber], Algaemax Wafers, New Life Spectrum, Homestead, FL, USA) five days per week in addition to grazing on benthic algae in the system. Broodstock were conditioned on the prepared diet for 2 months before successful spawning occurred.

### Spawning and fertilization

Group spawning was thermally induced following the methods described in Leber et al. (2009) and Moe (2014). One broodstock urchin died of an undetermined cause in the period after quarantine before spawning began. At each spawning attempt all (n = 13) broodstock were transferred to a 122-cm diameter polyethylene tank filled with 150-L of 1-μm filtered ASW. Water was heated to ~ 5 °C above holding tank temperature and supplemental aeration was provided. As broodstock spawned, gametes were collected using 60-mL catheter syringes and transferred to a separate container with 1-μm filtered ASW. Time to first spawn, as well as the number of male and female animals spawning were recorded. If a male spawned prior to a female, sperm was not collected and instead allowed to diffuse throughout the tank. The residual sperm concentration in the spawning tank allowed for fertilization to occur as eggs were collected and transferred. If a female spawn occurred prior to a male, eggs were collected in the same manner, however an unquantified amount of sperm was then collected upon release from a male and transferred to the egg container for mixing and fertilization. Embryos were then transferred to a climate-controlled room where they remained in the mixing container for 1–2 h while water temperature cooled to match the larviculture system (25–27 °C). Following cooling, embryos were moved to a single 40-L acrylic larviculture tank containing 1-μm filtered ASW and suspended in the water column using pulsed aeration. Two hours after fertilization, 1-mL subsamples were counted using a Sedgewick Rafter cell to estimate total egg count and fertilization rate.

### Recirculating aquaculture system

**Figure 2:**
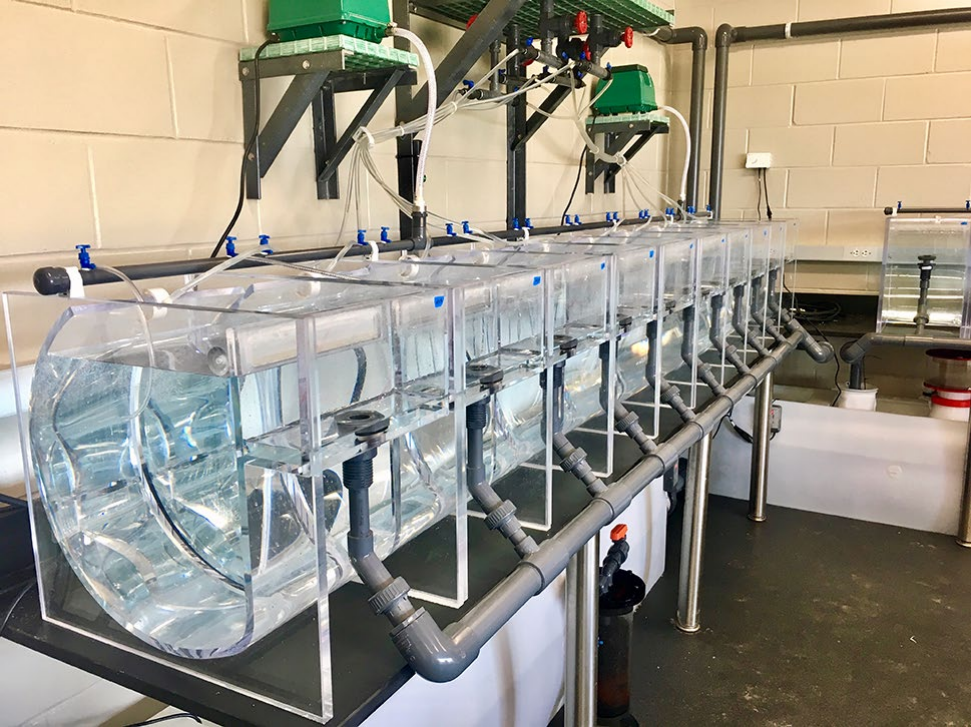
Photograph depicting configuration of the 1800-L recirculating larviculture system used to rear *D. antillarum* larvae.

At three days post-fertilization, early pluteus larvae were stocked into custom engineered 40-L acrylic culture tanks (details provided in supplementary materials) at densities between 1 and 2 larvae mL^−1^. The 40-L culture tanks were based on a design developed over fifteen years of experimental *D. antillarum* larviculture^49,50^. Aeration was supplied to individual culture tanks via air-wands positioned at the base of the vertical surface and pulsed at intervals of 3–5 s ON, and 20–30 s OFF using relay timer modules. The aeration timing was adjusted as necessary throughout larval development to prevent larvae from dropping out of suspension as they increased in size. Culture tanks were integrated within a novel 1800-L RAS designed to culture *D. antillarum* larvae while facilitating replicated experiments (Fig. 2; system schematic provided in supplementary materials). The RAS was located in a room with large north facing windows that provided a natural photoperiod. System life support components consisted of foam fractionators, ultraviolet sterilizers, ceramic biological filtration media, fluidized media reactors with activated carbon and granular ferric oxide, 5- and 25-µm mechanical filtration, and submerged heaters and chillers for precise temperature control. Twenty individual 40-L culture tanks were supplied with gravity-fed water from 150-L in-line header tanks at a flow rate of 1–2 L min^−1^. Culture tank water then passed through 105-µm mesh attached to an overflow weir, which allowed waste and unconsumed food to be flushed out while larvae were retained. Organic material and waste accumulation in the culture tanks necessitated infrequent maintenance during the culture period. During maintenance, larvae were siphoned into a submerged 105-μm sieve and then gently rinsed and transferred to a clean culture tank. Periodically, 10-mL subsamples were collected from culture tanks and counted to estimate larval density.

### Water quality

Approximately 30% of total system water volume was exchanged weekly using 1-µm filtered ASW. Water quality parameters including salinity, pH, total alkalinity, calcium (Ca^2+^), and phosphate (PO_4_^3−^) were monitored biweekly with a Hach DR 3900 spectrophotometer (Hach, Loveland, Colorado, USA). Ammonia (NH_3_-N) was monitored intermittently using the same spectrophotometer. Temperature was monitored daily. Salinity was also monitored multiple times weekly using a handheld meter (YSI Inc., Yellow Springs, OH, USA) and values were adjusted to a target of 35 g/L. Additional water samples were periodically sent to a commercial lab (Triton GmbH, Düsseldorf, Germany) for dissolved metal and total organic carbon (TOC) analyses via inductively coupled plasma optical emission spectrometry.

### Feeding regime and maintenance through larval development

**Figure 3:**
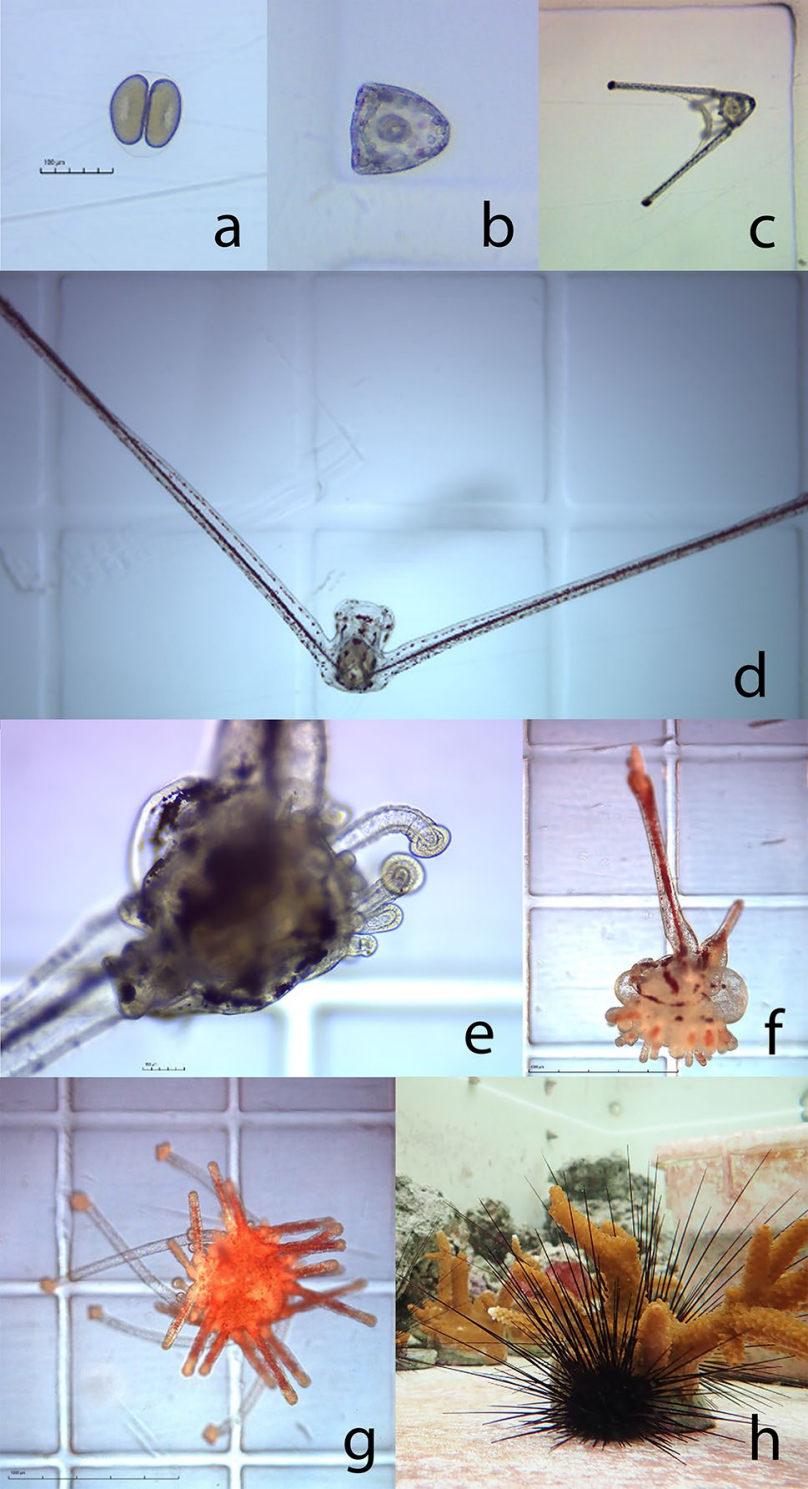
Representative photographs of *D. antillarum* development at (**a**) 2 h post fertilization, first cell division, D = 80 µm, (**b**) 36 h post fertilization, late gastrula/prism, mid body length (MBL) = 85 µm, (**c**) 3 d post fertilization, early pluteus larvae, MBL = 90 µm, appendage length (AL) = 190 µm, (**d**) 21 d post fertilization, 4-armed echinopluteus transversus larvae, MBL = 250 µm, AL = 2000 µm, (**e**) 28 d post fertilization, metamorphically competent late pluteus larvae with adult rudiment and extended tube feet, MBL = 600 µm, (**f**) 36 d post fertilization, mid-metamorphic radially symmetrical juvenile resorbing bilaterally symmetrical larval structure, D = 850 µm, (**g**) 36 d post fertilization, settled juvenile, D = 900 µm, (**h**) 248 d post fertilization, hatchery reared *D. antillarum,* D = 1–3 cm.

Live microalgae strains (the cryptophyte *Rhodomonas lens* and diatom *Chaetoceros gracilis*) were purchased from a commercial supplier (AlgaGen LLC, Vero Beach, FL, USA) and maintained in 18-L sterile carboys. Microalgae was first offered to larvae at 3 days post-fertilization (DPF). The feeding regime throughout the culture period consisted of a single feeding of live microalgae dosed daily to each culture tank at 17:00 followed by a feeding period during which no water was supplied from the header tanks. The following day at 09:00, water supply from the header tanks was re-initiated and the culture tanks were flushed for 8-h at a flow rate resulting in ~ 12–24 total tank turnovers. Various combinations of all three algal species were fed. Total algal cell densities were increased throughout the culture period as follows: 3 to 14 DPF (5–10 thousand cells mL^−1^), 14 to 28 DPF (15–20 thousand cells mL^−1^), and 28 DPF through metamorphic competency (30–40 thousand cells mL^−1^). Larvae were considered metamorphically competent after the appearance of an adult rudiment appendage and well-defined tube feet (Fig. 3e).

### Settlement and metamorphosis

The process by which planktonic larvae utilize adult tube feet to adhere to a benthic substrate and transition to a benthic life history is described as settlement^51,52^. Prior to settlement, bacterial and diatom biofilms were allowed to accumulate on culture tank surfaces. This method emulates existing urchin production practices, in which benthic biofilms are used to provide both a larval settlement cue and diet for newly settled juveniles^52–54^. At larval competency, pre-conditioned ceramic tiles from broodstock holding tanks were additionally placed in larval culture tanks in an attempt to provide additional settlement cues. The tiles contained an assemblage of potential cues including crustose coralline algae, benthic algae, and bacterial associates. After juveniles settled in the culture tank and grew to approximately 1-mm in test diameter, they were transferred to separate 55-L glass tanks with pre-conditioned live rock and ceramic tiles, benthic biofilms, and macroalgae (*Gracilaria* spp.).

## Results

### Spawning and fertilization

From 16 May 2018 to 25 August 2020, a total of 22 spawns were attempted. Eleven successful spawns are reported in Table 1 and were defined as those producing a minimum of 500,000 viable embryos. Eight spawn attempts were unsuccessful and complete data were not collected on three successful spawns. These are omitted from Table 1. All spawning attempts used the same common broodstock pool. For successful spawns, water temperatures were increased by 5.4 ± 0.3 °C (mean ± SEM) relative to the holding system temperature. Time from thermal induction to first gamete release averaged 3.6 ± 0.9 min. The proportion of individuals that spawned during each successful event was variable, ranging from 15.4 to 53.9% and averaging 34.3 ± 4.1%. The number of eggs extracted from each spawn ranged from 1.8 to 15.3 million with an overall mean of 5.0 ± 1.2 million. Sperm concentrations were not quantified, but fertilization rates were high and consistent at 94.0 ± 1.2% and no evidence of polyspermy was observed. While successful spawning events occurred throughout the calendar year, weak signals for both annual and lunar spawning patterns were observed. Simple linear regression revealed that the proportion of animals spawning in a given attempt was positively correlated with months relative to June (F (1, 20) = 9.80, *p* < 0.01), and negatively correlated with days since a new moon (R^2^ = 0.20, F (1, 20) = 5.12, *p* < 0.05).

**Table 1:** Date and associated data from 11 successful spawning events between 20 August 2018 and 25 August 2020 using a common pool of 13 *D. antillarum* broodstock.

Spawn date	Holding temperature (°C)	Spawn temperature (°C)	# Females spawning	# Males spawning	% of population spawning	Millions of eggs extracted (mean ± 95 CI*)	% Fertilization rate (mean ± 95 CI*)
20/8/18	26.0	31.0	2	5	53.8	2.2 ± 0.6	89.5 ± 2.1
17/9/18	26.2	31.0	2	3	38.5	15.3 ± 2.3	94.2 ± 3.7
5/10/18	26.3	31.0	2	1	15.4	1.8 ± 0.4	98.5 ± 0.4
7/11/18	25.0	30.0	3	4	53.8	4.7 ± 0.2	94.9 ± 5.4
18/2/19	23.9	30.0	2	2	30.8	3.8 ± 0.9	90.2 ± 2.7
21/5/19	26.8	31.0	1	1	15.4	9.3 ± 1.5	99.9 ± 0.3
18/9/19	27.8	34.5	3	1	30.8	3.7 ± 0.2	96.4 ± 0.5
29/10/19	26.7	31.0	1	4	38.5	2.7 ± 0.3	95.3 ± 1.7
29/1/20	23.9	31.0	3	3	46.2	5.8 ± 0.7	93.3 ± 4.6
12/6/20	26.3	31.7	2	1	23.1	3.9 ± 1.2	86.3 ± 14.2
25/8/20	25.9	32.3	3	1	30.8	1.9 ± 0.4	95.8 ± 1.8

Number of eggs extracted and fertilization rates reflect a 95% confidence interval (n = 3 counts per range).

*CI confidence interval.

### Water quality and larval development

All water quality parameters excluding ammonia are reported in Table 2. Ammonia was consistently undetectable. The mean concentrations of several dissolved metals known to have adverse biological effects on sea urchin larvae, including copper, nickel, selenium, lead, and zinc^55,56^ are also reported in Table 2.

**Table 2:** Mean ± SE water quality parameters monitored throughout the larval culture period.

Parameter (units)	Value (mean ± SEM)	n
**Water quality parameters**		
Salinity (g L_−1_)	36.09 ± 0.22	22
pH	8.15 ± 0.01	22
Alkalinity^a^ (mg L_−1_)	129.27 ± 2.46	22
Ca^2+^ (mg L_−1_)	425.28 ± 9.53	18
PO_4_^3−^ (mg L_−1_)	0.032 ± 0.01	21
TOC^b^ (mg L_−1_)	4.14 ± 1.17	3
**Dissolved metals**		
Cu (µg L_−1_)	1.50 ± 1.20	8
Ni (µg L_−1_)	0.78 ± 0.30	8
Se (µg L_−1_)	Undetectable	8
Pb (µg L_−1_)	Undetectable	8
Zn (µg L_−1_)	2.66 ± 1.45	8

*SEM* Standard error of the mean.

^a^Reported as CaCO_3_.

^b^Total organic carbon.

Periodic larval sampling throughout the culture period was conducted to document morphological development over time and is depicted in Fig. 3 (panels a–e). Viable eggs at 2 h post fertilization ranged from 70 to 80-μm in diameter. Development from the prism stage through early pluteus and the 4-armed stage, described as *Echinopluteus transversus* by Mortensen (1921), proceeded similarly to other members of the Diadematoida order^57–59^. Larvae at 21 DPF were heavily pigmented and had characteristically long fenestrated postoral arm appendages reaching ~ 2-mm in length. A period of high larval mortality indicated by body condition deterioration and declines in larval density was observed in multiple culture attempts at 28–35 DPF. Larvae that survived these mortality events, which averaged < 1.5%, developed adult rudiment appendages and well-defined tube feet at 28–93 DPF, indicating metamorphic competence.

### Metamorphosis and settlement

Figure 3 (panels f–h) depicts larval settlement and juvenile urchin development. Following the addition of pre-conditioned ceramic tiles, competent larvae appeared to initiate settlement on biofilm surfaces within the culture tank and also on the tiles themselves. Sampled juveniles undergoing settlement were observed to resorb remnant larval appendage structures as they transitioned from bilaterally symmetrical plankton to radially symmetrical benthic urchins. Newly settled juveniles had orange and red tests and spines under top illumination and did not appear to have yet developed an Aristotle’s lantern feeding apparatus. Juvenile growth rates were highly variable despite low animal densities and high resource availability in the 55-L glass tanks. The most successful culture attempt resulted in a total of 100 juveniles surviving past 90 DPF from a single 40-L culture tank. This represents an 8.3% post-settlement survival rate from 1200 competent larvae, and a ~ 0.125% overall survival rate from the initial stocking of 80,000 larvae at 3 DPF. By 60 days post settlement, most juveniles appeared entirely black in coloration. Individual test diameters at 248 DPF ranged from 1 to 3 cm. Delayed settlement in subsequent successful culture attempts occurred at 60–93 DPF with a maximum of 49 juveniles produced per attempt.

## Discussion

The successful culture process outlined in this study improves the viability of *D. antillarum *ex situ production for potential population enhancement as a means to restore yet-to-recover Caribbean herbivory. While this species has been cultured before^48–50^, knowledge gaps and impediments in the culture process have resulted in limited success. Chief among these impediments was the inability to repeatedly rear larvae to the point of metamorphic competency and settlement in a culture tank capable of scaled production^49^. While other urchin species are cultured at scale for commercial food markets^60^ and even coral reef restoration^61,62^, unique *D. antillarum* larval biology has prevented existing production methods from being applied and has necessitated a novel approach. The culture success described in this study was contingent on (1) a reliable non-invasive spawning procedure, (2) a unique larviculture system design, and (3) reproducible larval rearing protocols. These methods have advantages and limitations, especially in the context of potential future *D. antillarum* population enhancement.

Commercial and research-oriented echinoderm aquaculture typically employs dissection or coelomic KCl injection to extract gametes or induce broodstock spawning^53,63,64^. Given the scarcity of *D. antillarum*^32^ and general broodstock mortality and poor egg quality associated with dissection and coelomic injection^50,65,66^*,* a less destructive spawning method is preferred for this species. Capo et al. (2003) investigated the potential for constant photo-thermal parameters to support spontaneous spawning for year-round scaled *D. antillarum* hatchery production. Within the current system, a desire for on-demand gamete collection prompted application of thermal induction group spawning^50^. The method used by Capo et al. (2003) produced on average 89,000 eggs per spawn across 173 spontaneous spawning events over 19 months. The present method resulted in at least 11 successful induced spawning events over 24 months that produced on average five million eggs per spawn. It is important to note that, because gametes were collected using a syringe from actively spawning and submerged females, egg numbers reported here likely underestimate total fecundity. Further, the overall spawning success rate using the thermal induction was ~ 64% (14 successes in 22 attempts). This presents room for improvement and based on subsequent observations, we speculate that a constant broodstock holding temperature of ~ 26 °C may contribute to more reliable spawning. During this study, broodstock holding system temperatures were programmed to fluctuate seasonally, with a maximum observed water temperature of 28.8 °C. While this temperature is well within those expected in the natural range of *D. antillarum*, it should be noted from Table 1 that only one successful spawning event occurred when broodstock holding system temperature exceeded 27.0 °C. After this study, broodstock management practices have shifted to a constant holding temperature of ~ 26 °C and spawning has anecdotally become more reliable. Spawning success could additionally be impacted through diet^67^ and photoperiod^68,69^ manipulation. While thermal induction also results in more partial or incomplete spawns compared to coelomic injection^66^, this method did work effectively to produce large numbers of fertilized embryos without causing undue broodstock mortality. Importantly, this method also allows determination of the number of contributing broodstock and their sex. Group thermal induction, however, resulted in relatively low proportions of the total broodstock pool contributing gametes. Similar observations have been reported in other urchin species^66^. Thus, strategies to mitigate undesirable genetic impacts will be critical for the full realization of scaled *D. antillarum* production for responsible stock enhancement^70^.

Precautionary stock enhancement paradigms suggest that broodstock should be of local origin and that effective population sizes should be maximized in the hatchery so as to prevent deleterious genetic intrusions into wild populations^70^. Chandler et al. (2017) examined the genetic diversity of wild *D. antillarum* populations from Biscayne Bay to the Dry Tortugas alongside two locally sourced captive broodstock populations. This study concluded that little genetic differentiation existed among wild and broodstock populations and that captive bred Florida urchins would sufficiently reflect the diversity of wild populations^71^. While this suggests that hatchery production and restocking could viably be used to restore *D. antillarum* populations throughout the studied range with little risk of outbreeding depression, large numbers of restocked offspring progenerated from few broodstock could still pose risks to the genetic structure (i.e. effective population size) of endemic populations as they interbreed with wild individuals^70,72, 73^. In the present study, the proportion of spawning *D. antillarum* was sometimes as low as 15.4%, with genetic contributions from only one male and one female. Thermal induction resulted in a low proportion of spawning individuals and, if used for scaled production and restocking, would necessitate housing and spawning an extensive number of broodstock in order to attain a minimal effective hatchery population size. Conducting future hatchery cohort parentage analyses could help to refine this approach. Avoiding potentially deleterious genetic intrusions will alternatively require targeted research aimed at increasing broodstock contributions to hatchery cohorts. If reliably high yields of juvenile urchins are attained from future larviculture attempts, revisiting the use of established yet destructive methods to enhance gamete extraction from broodstock could be justified. These methods may mitigate genetic risks inherent to a restocking program by improving hatchery genetic diversity.

Further investigations into adult urchin nutrition and other factors affecting spawning success could further refine broodstock management practices for scalable production. While specialized diets have been shown to improve gonad production and quality^53,74–76^, few commercial products exist and none have been tailored specifically for tropical urchin aquaculture^64^. The generic herbivore diet used in this study produced consistently high numbers of viable embryos from spawning broodstock. However, more research could help to optimize a diet to further improve the proportion of spawning individuals, gonad production and quality, and even larval success^77,78^. Several environmental factors including but not limited to lunar periodicity, water temperature, and photoperiod, may also influence *D. antillarum* spawning patterns and success^79–81^. Studies examining spawning patterns in wild populations have produced conflicting results, with some suggesting strong synchrony with annual and lunar cycles^82–84^ and others describing less predictable patterns^85–87^. Such discrepancies may be due to latitudinal gradients among populations^81^. In this study, instances of successful spawning events occurred throughout most of the calendar year, suggesting that the described broodstock management practices can be utilized for year-round hatchery production. However, the proportion of spawning broodstock was generally higher during the Spring and Fall months, which supports observations of peak spawning periods in several wild populations^83,84^. The apparent positive correlation between broodstock spawning proportion and the presence of a new moon suggests that additional factors including lunar periodicity affected spawning performance in captivity. This is supported by several observations of wild spawning patterns at or around the time of the new moon^83–85^. Broadly, both seasonal and lunar cycles appeared to influence spawning performance in this study; however, these conclusions are uncertain given the small sample size. In situations where constant gamete production would be preferred, it could be conceivable to reduce cyclical spawning patterns by subjecting broodstock to constant photo-thermal parameters as described by Capo et al. (2003).

Compared to commonly cultured echinoderm larvae, which possess up to four paired arm structures, *D. antillarum* larvae have only two paired arms with one greatly elongated postoral pair (see Fig. 3d). Because of their distinct *Echinopluteus transversus* morphology, *D. antillarum* larvae are considerably less robust than other echinoderm larvae and more susceptible to mechanical breakage^49,88^. Further, while echinoderm larvae are negatively buoyant and generally poor swimmers^60,89^, diadematid larvae are particularly ineffective at self-propulsion^57^ and likely exhibit low stability in turbulent systems^49,90^. *D. antillarum* larvae cultured in this study were observed to sink and die without adequate flow. These factors necessitated applying novel flow dynamics to the culture tank such that larvae remained physically suspended while minimizing breakage^49,50^. The unique geometry of the 40-L culture tank was critical to providing this environment. High initial stocking densities might also adversely affect the survival of long-armed larvae^88^. Therefore, *D. antillarum* larvae were stocked at 1–2 larvae mL^−1^ compared to densities upwards of 10 larvae mL^−1^ used successfully in species such as *Tripneustes gratilla*^91^. A diadematid urchin (*Centrostephanus rodgersii*) with similar larval morphology to *D. antillarum* was successfully cultured in 125-L and 300-L tanks at final larval densities of 0.2–1.0 mL^−1^ ^92,93^. The 300-L tanks were cylindro-conical and “gently aerated”^93^. Such a configuration has been anecdotally tested with *D. antillarum* in smaller tanks and resulted in larval mortality. While it is possible that increasing tank size could generate different results, it appears that *D. antillarum* larvae may be more negatively buoyant and less motile than *C. rodgersii*. Similarly, the successful culture of this species in larger versions of the tank described here is unknown, yet feasible. Larger culture tanks with comparable geometry, flow rates and turbulence have the potential to further enhance larval production. However, it may not be necessary to do so as overcoming larval mortality bottlenecks within the system described could result in production of adequately high numbers of competent larvae from each tank.

While aspects of prior successful culture methods were incorporated into this study^48,49^, considerations were made to account for known issues of scalability and culture bottlenecks. The system design was intended to balance the desire for large-scale production with a concurrent need for replicated aquaculture research. Conventional small-scale urchin culture methods rear larvae in 2- to 4-L beakers for ecotoxicology and developmental biology research^60,88^. The novel RAS described here incorporated 40-L culture tanks capable of rearing up to 5000 competent larvae in each tank. Larger vessels with a similar geometry could conceivably be designed to rear larger quantities of *D. antillarum*. Smaller tanks, however, provide the opportunity to reduce labor and increase experimental replication to resolve culture bottlenecks. Prior *D. antillarum* culture success^49,50^ was accomplished in standalone 50-L prototype culture tanks that required frequent cleaning and intensive labor for maintenance, thereby limiting replication. The methods described here resolved this problem by incorporating up to twenty culture tanks into an RAS with robust life support components that reduced the need for cleaning and water exchange. This allowed for greatly reduced labor and increased replication needed for rigorous hypothesis testing. Preventing larval waste products from accumulating and negatively affecting water quality in the culture tank is also essential for rearing echinoderm larvae^88^. Because *D. antillarum* larvae are mechanically fragile, standard methods used to directly remove waste from culture vessels can cause mortality from breakage. The ability to flow treated recirculating water through the culture tanks mitigated the accumulation of waste products. Another consideration for system design was the necessity to minimize larval exposure to dissolved metals.

Sea urchin larvae are often used as bioindicators for dissolved metal pollution due to extreme susceptibility^94,95^. *D. antillarum* larvae are among the most sensitive marine organisms to dissolved metals, with abnormal development occurring at nickel, selenium, silver, and copper concentrations as low as 15 µg L^−1^, 26 µg L^−1^, 6 µg L^−1^, and 11 µg L^−1^, respectively^55^. By comparison, purple sea urchin, *Stronglyocentrotus purpuratus,* larvae are sensitive to dissolved nickel concentrations of 250 µg L^−1^ ^95^. Bivalve species in the genus *Mytilus* are the only aquatic taxa found to be more sensitive to dissolved silver and copper than *D. antillarum*^96,97^. Nickel may be a particularly prevalent trace metal found in aquaculture systems^98^, potentially due to the use of submerged stainless-steel components in pumps and other hardware. Metal toxicity was a suspected factor in failed metamorphic development for previous *D. antillarum* culture attempts^49^. The use of filtered natural seawater may also introduce unknown contaminants. Difficult to remove contaminants including dissolved metals or toxic organic compounds found in source waters may negatively impact larval development and successful metamorphosis^99^. The potential for contaminated source water was minimized by using artificial saltwater comprised of purified freshwater and a high-quality salt mixture. Dissolved metal concentrations were kept below known thresholds through weekly water changes, addition of metal adsorbing materials including granular ferric oxide and Poly Filter (Poly-Bio-Marine Inc., Pennsylvania, United States), and a specially formulated, low dissolved metal F/2 microalgae growth media. Future culture attempts could benefit from additional filtration methods to reduce dissolved metal concentrations. Standard water quality parameters were otherwise optimal.

The algal feeding regime used was similar to that of Eckert (1998) and Leber et al. (2009). The microalgae species common to all successful culture attempts was the cryptophyte, *Rhodomonas lens*^48–50^, which has a high fatty acid, protein, and amino acid concentration^100^. This microalgae species also has a large cell size relative to other commercially available microalgae. Pigment content in *R. lens* might partially explain larval culture success due to various biological functions including photoprotection, immunological response, and antioxidant activity^101,102^. Carotenoid content can also influence egg production and development, and disease response when included in the diet of adult sea urchins^76,100,103^. Mixed-algae diets were used because they have been shown to improve larval development in other urchin species^78,104^. Developing a feeding regime required balancing recirculating flow-through rates to sustain water quality with sufficient duration of larval exposure to algal cells for adequate feeding. A 16-h static feeding period followed by 8 h of flow comprising 12–24 culture vessel turnovers was determined to meet this requirement. Constant flow-through would provide better water quality and could possibly mitigate the risk of toxin accumulation, bacterial infection, and larval mortality^105^. However, constant flow-through would also flush uneaten microalgae out of the larviculture tank and might result in diminished larval growth.

Larvae settled as early as 36 DPF after the addition of pre-conditioned ceramic tiles, which is within the range of prior successful culture attempts^48,49^. Sea urchin larval settlement is a dynamic process and can be impacted by a multitude of factors including available biochemical cues^54,106^, mechanical cues^107^, and nutritional status^78^. Further, post-settlement survival is a major bottleneck for many marine invertebrates^108^ and represents a major challenge to commercial urchin aquaculture efforts^54^. Sea urchin larvae must be nutritionally competent in order to survive the period between settlement and development of the juvenile gut and Aristotle’s lantern feeding apparatus, which are needed for exogenous feeding^60,109, 110^. The underlying dynamics affecting *D. antillarum* larval settlement and post-settlement survival should be the subject of future research. Even moderate improvements in these areas will drastically improve the feasibility for mass production to meet restoration objectives. Further elucidation of settlement dynamics in the laboratory would also provide insight into the factors affecting settlement and recruitment in the wild and could improve our understanding of the limited *D. antillarum* natural recovery.

While the focus of this study involved generating reliable ex situ aquaculture methods for *D. antillarum,* it should be noted that the restocking strategy illustrated in Fig. 1 is only necessary absent natural population recovery to historical abundances. The goal of this strategy is reestablishment of self-sustaining urchin populations and associated herbivory. However, the same ecological factors limiting natural recovery will also likely challenge future restocking attempts. It is therefore necessary to consider in situ barriers to recovery and how these barriers might impact restocking success. Drastic reductions in fertilization success and low larval supply following mass mortality events in 1983 and 1991 are theorized to be one of the major factors limiting recovery in the Florida Keys^111,112^. Enhanced *D. antillarum* populations could potentially alleviate some of this limitation through increased gamete production (genetic concerns discussed above should be considered). Evidence exists, however, to suggest that post-settlement limitations are equally important in recovery dynamics. Despite largely recovered recruitment rates in Curaçao between 1984 and 2005, only modest increases in adult abundances implied high levels of post-settlement mortality^113^. Likewise, moderate adult densities in Puerto Rico were observed in areas with low larval settlement rates^112^. One possible explanation includes positive density-dependent mechanisms whereby juvenile recruitment and survival increases in the presence of adult urchins^114,115^. Other studies reason that habitat complexity is crucially important for mediating *D. antillarum* distributions due to the availability of predation refuge^47,116, 117^. Ironically, the loss of habitat complexity due to increased macroalgae and decreased hard coral cover likely constrains *D. antillarum* recovery. Negative feedback mechanisms such as this, along with reduced settlement habitat due to increased macroalgae cover^39^, work to stabilize macroalgae states over the long-term^118^. All factors considered, the efficacy of future hatchery restocking is unknown and extensive experimentation is advised. Initial attempts to augment *D. antillarum* populations in the Florida Keys and Curaçao through translocation have highlighted the need to concurrently provide artificial shelter^119,120^. One population recovery model postulated that restocking urchins large enough to escape predation pressure will be a necessary initial step towards phase shift reversal^121^. Tandem restoration with reef-building hard corals should be explored as a potential solution to both provide predation refuge for urchins and improve multi-niche survival outcomes.

Reducing macroalgae cover and improving ecological conditions that favor Western-Atlantic and Caribbean hard coral recruitment via *D. antillarum* population enhancement could be a powerful tool to augment existing reef restoration goals. To date, knowledge gaps and limited research, in part due to a lack of commercial interest^122^, have impeded the ability to reliably culture *D. antillarum* at scales sufficient to attempt this strategy^121^. The system described here incorporates tractable broodstock management and spawning, a RAS that balances requirements of a unique larval biology with the potential for experimental replication and scalable production, and a successful larviculture protocol. Further improvements to *D. antillarum* production within this system will necessitate an understanding of unknown bottlenecks causing larval mortality. The absence of observable water quality problems suggests that the most important issues facing the described system related to larval nutrition and/or disease. Continued culture attempts alongside strategic investigations into improved microalgal diets and disease dynamics will be necessary for further improving the viability of production for restoration. Although much more work is required before *D. antillarum* culture for population enhancement becomes a reality, the establishment of a balanced system design with reproducible results is an encouraging step forward.

## Supplementary information

Supplementary Information.
