# Supplementary material for: A novel system for intensive Diadema antillarum propagation as a step towards population enhancement
Source: Sci Rep. 2021 May 27;11:11244. doi: 10.1038/s41598-021-90564-1 (PMC8160213; doi:10.1038/s41598-021-90564-1)
Supplement: Supplementary file 1 — Supplementary Information. [file 41598_2021_90564_MOESM1_ESM.pdf]

A novel system for intensive *Diadema antillarum* propagation as a step towards population enhancement

Aaron R. Pilnick[1*], Keri O'Neil[2], Martin Moe, Joshua T. Patterson[2,3]

[1]Interdisciplinary Ecology, School of Natural Resources and Environment, University of Florida/IFAS 103 Black Hall, Gainesville, Gainesville, FL 32611, USA
[2]Center for Conservation, The Florida Aquarium, 529 Estuary Shore Lane, Apollo Beach, FL 33572, USA
[3]Fisheries and Aquatic Sciences, School of Forest Resources and Conservation, University of Florida/IFAS 7922 NW 71st Street, Gainesville, FL 32603, USA
*apilnick@ufl.edu

*Communicating Author: Aaron Pilnick apilnick@ufl.edu

Electronic Supplementary Material

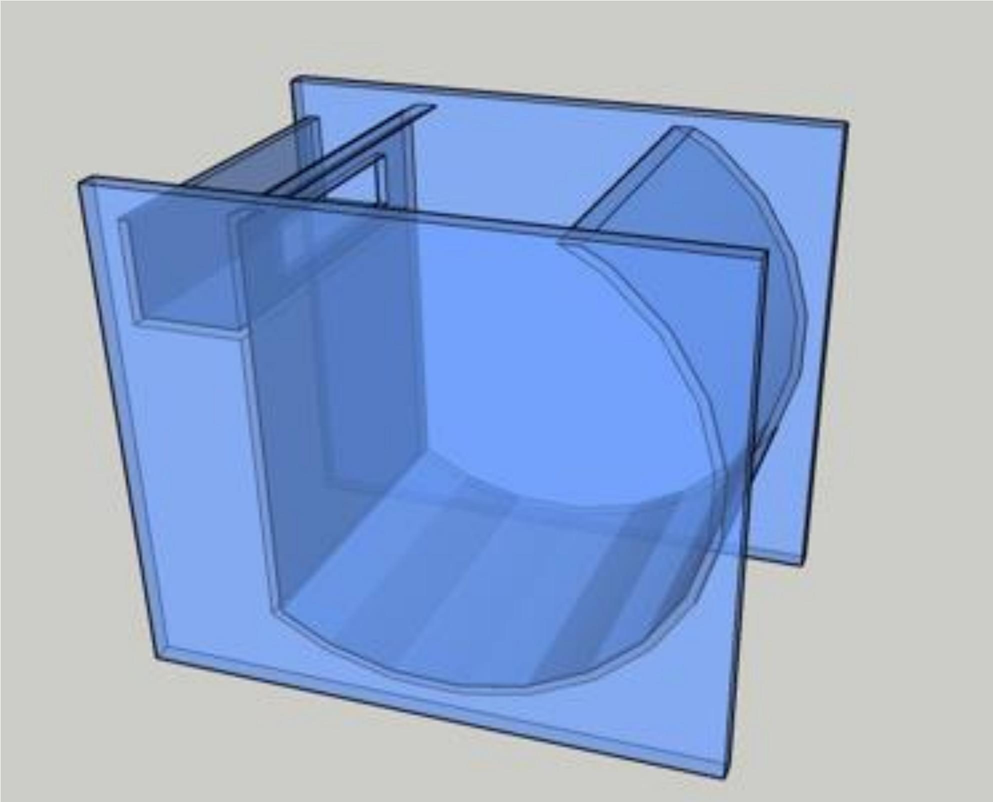

1.a Sketchup design of the 40-L larviculture tank

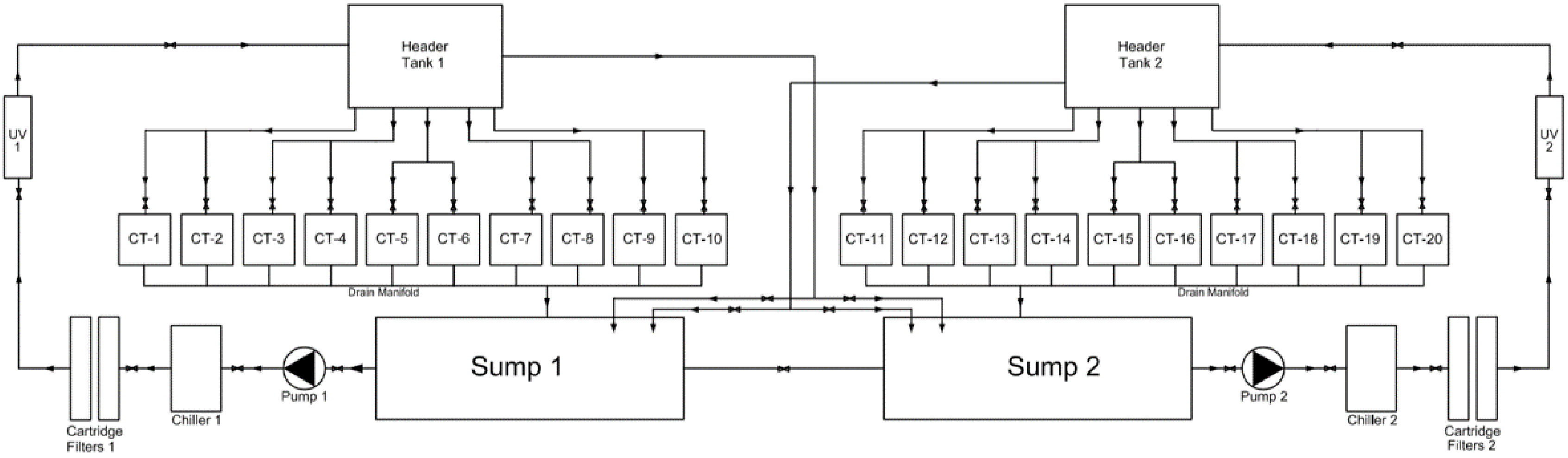

1.b Schematic of the 1800-L larval culture system. Figure by Chris Nguyen.
